# Nuclear Disposition of Alien Chromosome Introgressions into Wheat and Rye Using 3D-FISH

**DOI:** 10.3390/ijms20174143

**Published:** 2019-08-25

**Authors:** Veronika Koláčková, Kateřina Perničková, Jan Vrána, Martin Duchoslav, Glyn Jenkins, Dylan Phillips, Edina Turkosi, Olga Šamajová, Michaela Sedlářová, Jozef Šamaj, Jaroslav Doležel, David Kopecký

**Affiliations:** 1 Institute of Experimental Botany of the Czech Academy of Sciences, Centre of the Region Haná for Biotechnological and Agricultural Research, Šlechtitelů 31, 78371 Olomouc, Czech Republic; 2 Department of Botany, Faculty of Science, Palacký University Olomouc, Šlechtitelů 27, 783 71 Olomouc, Czech Republic; 3 Institute of Biological, Environmental and Rural Sciences, Aberystwyth University, Aberystwyth, Ceredigion, Wales SY23 3DA, UK; 4 Agricultural Institute, Centre for Agricultural Research, Hungarian Academy of Sciences, H-2462 Martonvásár, P.O. Box 19, Hungary; 5 Department of Cell Biology, Centre of the Region Haná for Biotechnological and Agricultural Research, Faculty of Science, Palacký University Olomouc, Šlechtitelů 27, 783 71 Olomouc, Czech Republic

**Keywords:** 3D-FISH, barley, chromatin, hybrid, introgression, nucleus, rye, wheat

## Abstract

During interphase, the chromosomes of eukaryotes decondense and they occupy distinct regions of the nucleus, called chromosome domains or chromosome territories (CTs). In plants, the Rabl’s configuration, with telomeres at one pole of nucleus and centromeres at the other, appears to be common, at least in plants with large genomes. It is unclear whether individual chromosomes of plants adopt defined, genetically determined addresses within the nucleus, as is the case in mammals. In this study, the nuclear disposition of alien rye and barley chromosomes and chromosome arm introgressions into wheat while using 3D-FISH in various somatic tissues was analyzed. All of the introgressed chromosomes showed Rabl’s orientation, but their relative positions in the nuclei were less clear. While in most cases pairs of introgressed chromosomes occupied discrete positions, their association (proximity) along their entire lengths was rare, and partial association only marginally more frequent. This arrangement is relatively stable in various tissues and during various stages of the cell cycle. On the other hand, the length of a chromosome arm appears to play a role in its positioning in a nucleus: shorter chromosomes or chromosome arms tend to be located closer to the centre of the nucleus, while longer arms are more often positioned at the nuclear periphery.

## 1. Introduction

During interphase, eukaryotic chromosomes decondense and occupy distinct regions of the nucleus, named chromosome territories or chromosome domains [1,2]. Each chromosome territory (CT) is a complex structure of irregular shape and it appears to be largely stable during the interphase of the cell cycle [3,4,5]. Although CTs are spatially separated from each other by interchromosomal domains, there are regions where neighboring territories intermingle [6].

Chromosome painting, where whole chromosome probes are used for FISH of single CTs or groups of CTs, showed that interphase chromosomes are radially arranged in human [7,8] primates [9], chicken [10], and mouse [5]. This type of the CT arrangement appears to be evolutionary conserved among vertebrates [11,12]. However, the radial chromosome arrangement has not been observed in flowering plants. Instead, chromosome territories show two predominant configurations, which are known as Rabl’s and Rosette. In the Rabl’s orientation [13], centromeres are grouped at, or close to, the nuclear periphery at one pole of the nucleus, while telomeres are dispersed at the opposite pole [14]. This configuration is found in many large-genome plant species and is a remnant of anaphase chromosome movement [15]. A completely different type of nuclear organization has been observed in *Arabidopsis thaliana* (L.) Heynh., where the CTs exhibit rosette-like structures [16]. Centromeres are randomly distributed at the nuclear periphery, while telomeres congregate around the nucleolus. Centromeric heterochromatin forms distinct chromocenters, and euchromatin domains, where the majority of genes are located, create 0.2–2Mb loops that result in rosette-like structures of interphase chromosomes [16,17,18]. The position of CTs and the arrangement of heterochromatin domains is mostly random in differentiated as well as in meristematic tissues, except for chromosomes with nucleolar organizing regions (NORs), which associate with the nucleolus [16,19].

There is a growing evidence that links nuclear architecture with gene expression. Structural genes are often regulated by loci that are located far from the genes themselves, and sometimes even on different chromosomes. Methodological advances provided a way to map in detail the contacts between genes and their regulators, including promoters and enhancers. The method of chromosome conformation capture (3C) and its modifications, such as 4C and Hi-C, reveal contacts between chromatin fibres [20,21]. Using Hi-C, Dixon et al. [22] found that DNA within each chromosome territory is organized into chromatin domains, which can change their spatial conformation. Chromatin fibres within and between these topologically associated domains (TADs) can interact and affect gene expression via the formation of new contacts between enhancers and promoters, or by interrupting the existing contacts. These interactions are reorganized during growth and development, and in reaction to external factors [23,24].

Merging two genomes via interspecific hybridization may alter chromatin organization, and hence the interactions between genes and their regulatory elements. Gene expression studies on ancient and newly developed allopolyploids reveal substantial changes in gene expression [25]. However, the organization of parental chromatin in interspecific hybrids, as well as its effect on gene expression, remains poorly understood. In Chinese hamster × human hybrid cell lines, the human X chromosome (as the only human chromosome present) is located in a distinct chromatin body [26]. Similarly, Sengupta et al. [27] provided evidence of the stable positioning of gene-poor human chromosomes 7 and 18 on the periphery of human × mouse hybrid nuclei. These results indicate that the location of a CT in a hybrid nucleus might be affected more by its gene content rather than by its parental origin. Even less is known about nuclear architecture in the interspecific hybrids of plants. Chromosome arms of rye (*Secale cereale* L.) introgressed into bread wheat (*Triticum aestivum* L. ssp. *aestivum*) display a typical Rabl′s orientation and they appear to be located at the periphery of cell nuclei, while homologous arms are usually spatially separated from each other [28,29]. However, these findings were based on two-dimensional (2D) squash preparations in which the third dimension was compromised.

The studies on spatial CT organization in plants with large genomes, such as wheat, rye, and barley, are hampered by the lack of chromosome-specific painting probes for FISH. A solution is to use alien chromosome introgressions or substitutions and visualize alien chromosomes by genomic *in situ* hybridization (GISH) while using labelled genomic DNA from the donor species. This study employed this approach to shed more light on the spatial organization of parental genomes in the somatic nuclei of interspecific plant hybrids and to compare the nuclei from different cell cycle phases, various tissues, with contrasting relative proportions of parental genomes and varying the lengths of introgressed chromosomes (or arms). A combination of a wide set of various introgression lines (rye introgressions in wheat, barley in wheat and wheat in rye), 3D-GISH, confocal microscopy, and visualization of parental chromatin in software Imaris, made it possible to evaluate the potential impacts of the above-mentioned features on the position of the introgressed alien chromatin.

## 2. Results

### 2.1. Morphometric Characteristics of G_1_ Nuclei of Wheat-Rye and Wheat-Barley Chromosome Introgression Lines

**Figure 1:**
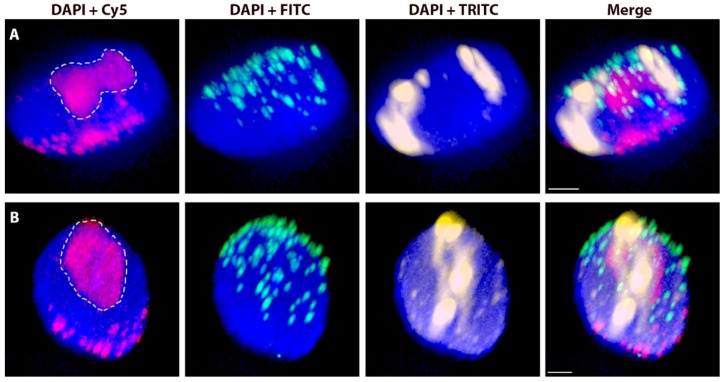
Nuclei (from root tips) with rye chromosomes in wheat background after 3D-FISH. (**A**) Nucleus with a pair of rye 1R chromosomes substituted for wheat chromosomes 1A. (**B**) Nucleus with a pair of rye _del_1RS.1RL chromosomes. Total genomic DNA of rye was labelled with TRITC using Nick translation (yellow), centromeres of both wheat and rye chromosomes were visualized using oligonucleotide probe (magenta), and telomere-specific sequence was PCR-labelled with FITC (green). Nuclear DNA was counterstained with DAPI (blue). Nucleoli areas are indicated by white dashed lines. Scale bar 3 µm.

**Figure A1:**
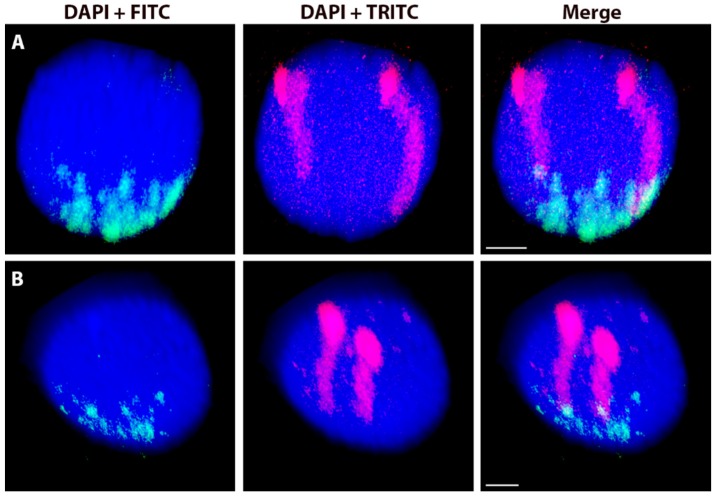
Wheat nuclei with a pair of homologous rye chromosome arms after 3D-FISH. A nucleus of 2BS.2RL (**A**) and 5RS.5BL (**B**) translocation lines. Total genomic DNA of rye was labelled with TRITC using Nick translation (magenta) and the centromere-specific sequence was PCR-labelled with FITC (green). Nuclear DNA was counterstained with DAPI (blue). Scale bar 3 µm.

**Figure A2:**
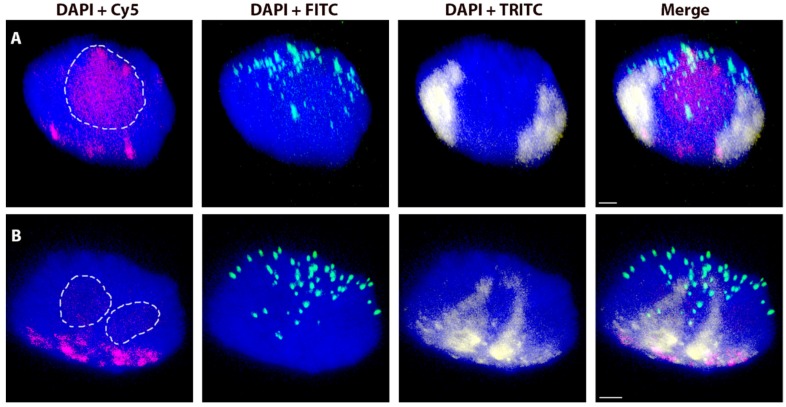
Rye nuclei with a pair of homologous wheat chromosomes. A nucleus carrying chromosome 3B (**A**) and chromosome 5B (**B**). Total genomic DNA of wheat was labelled with TRITC using Nick translation (yellow), centromeres of both wheat and rye chromosomes were visualized using oligonucleotide probe (magenta), and telomere-specific sequence was PCR-labelled with FITC (green). Nuclear DNA was counterstained with DAPI (blue). Nucleoli are indicated by white dashed lines. Scale bar 2 µm.

**Figure A3:**
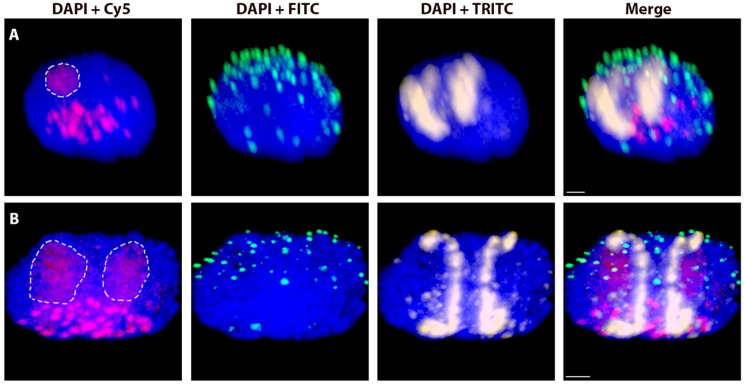
Wheat nuclei with a pair of homologous barley chromosomes. A nucleus carrying chromosome 3H (**A**) and chromosome 6H (**B**). Total genomic DNA of barley was labelled with TRITC using Nick translation (yellow), centromeres of wheat chromosomes were visualized using oligonucleotide probe (magenta), and telomere-specific sequence was PCR-labelled with FITC (green). Nuclear DNA was counterstained with DAPI (blue). Nucleoli are outlined by white dashed lines. Scale bar 3 µm.

In total, 481 G_1_ nuclei were analyzed, isolated from root tips of plants carrying rye or barley chromatin introgressed into wheat background or wheat chromatin introgressed into rye background (Figure 1, Figure A1, Figure A2 and Figure A3). The parameters that are described in Materials and Methods were evaluated. Generally, the morphology of the G_1_ nuclei, which were flow sorted into polyacrylamide gels, ranged from spherical and ellipsoidal to irregular shapes with varying degree of contortion. Only nuclei with spherical and slightly ellipsoidal shapes were selected for analyses. In 3D-GISH, the CTs of introgressed chromosomes or chromosome arms appeared as compact structures of regular shapes that were arranged in a typical Rabl’s orientation. Centromeres and telomeres of the host chromosomes also displayed Rabl’s configuration. Centromeres were generally close to each other and located at the nuclear periphery at one pole of the nucleus, while the telomeres were located at the opposite pole and usually dispersed over larger volume of the nuclei than the centromeres. In a majority of cases, the centromeres of introgressed chromosomes or chromosome arms were closer to each other than their telomeres and arm mid-points (MA). The distances between the arm mid-points and between their telomeres are similar in a majority of lines (Table 1).

Among all the analyzed nuclei, there are 315 G_1_ nuclei carrying various introgressions of rye chromatin into wheat background (Figure 1 and Figure A1). Volumes of these nuclei range from 753 to 3407 µm^3^ (average 1618 µm^3^). The lengths of individual rye chromosome arms ranged from 3.7 µm to 20.9 µm. For wheat chromosomes introgressed into rye background, 40 G_1_ nuclei were analyzed (Figure A2). Nuclear volumes range from 1029 to 2535 µm^3^ (average 1514 µm^3^) and lengths of individual wheat chromosome arms range from 1.8 to 12.2 µm. For barley introgressions into wheat background, the total number of analyzed G_1_ nuclei is 126 (Figure A3). The nuclear volumes range from 900 to 3872 µm^3^ (average 1721 µm^3^) and lengths of individual barley chromosome arms range from 1.4 µm to 15.1 µm. In lines 6H and 6HS, the nucleolus is frequently present on the short arm; the secondary constriction, which makes the arm longer: the average length of the 6HS arm, including the nucleolus is 6.7 and 4.1 in complete chromosome 6H and telocentric 6HS; when the nucleolus constriction is excluded from the total measurements, it is 4.2 and 3.5 µm for 6H and 6HS, respectively.

**Figure 2:**
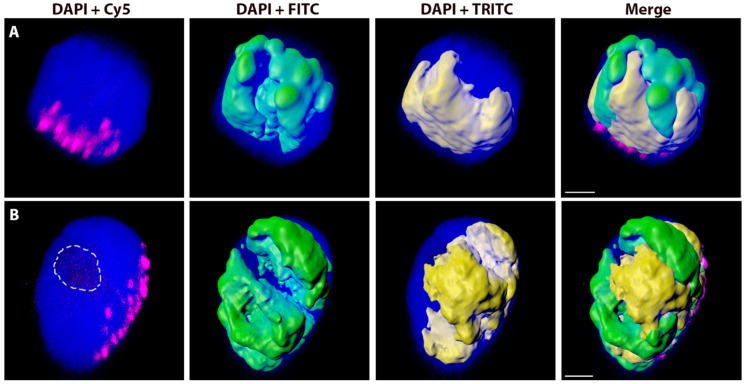
Nuclei of tetraploid (**A**) and hexaploid (**B**) triticale with labelled rye and wheat chromatin. Total genomic DNA of rye was labelled with FITC using Nick translation (green), total genomic DNA of wheat was labelled with TRITC (yellow), and centromeres of both wheat and rye chromosomes were visualized using oligonucleotide probe (magenta). Nuclear DNA was counterstained with DAPI (blue). Wheat and rye chromatin was visualized in Imaris 9.2 using the ‘Surfaces’ function. The nucleolus area is indicated by a white dashed line. Scale bar 3 µm.

In total, 44 G_1_ nuclei of triticale were analyzed (Table 2). The CTs of wheat and rye chromatin are readily distinguishable from each other and they do not appear to intermingle (Figure 2, Video S1). All of the chromosomes display Rabl’s configuration with their centromeres located close to each other at the nuclear periphery. The nuclear volume of tetraploid triticale ranged from 600 to 1587 µm^3^ (average 993 µm^3^). On average, 53.6% of this volume is occupied by rye chromatin, while 46.4% is occupied by wheat chromatin. The nuclear volume of hexaploid triticale ranged from 1097 to 2322 µm^3^ (average 1412 µm^3^), with 37.7% of the nuclear volume belonging to rye chromatin, while 62.3% is occupied by wheat chromatin. These values, for both ploidy levels in triticale, appear to be directly related to the relative DNA contents of the wheat and rye genomes.

### 2.2. Variation in the Spatial Arrangements in G_1_ Nuclei of Alien Chromosome Arms in Various Wheat-Rye and Wheat-Barley Introgression Lines

There appears to be a relationship between the spatial positioning of a rye chromosome arm and its length. The shortest complete rye chromosome arm, 5RS, tracks most frequently through the inner volume of the nucleus, while the longest arm, 2RL, is more frequently located on the periphery, close to the nuclear envelope. Chromosome arm length and the arm ratio on the spatial positioning of a chromosome arm, the distances between the mid-points of rye arms, and the centres of the nuclei (MA-CN) were compared, after standardization for the nuclear volume to test the possible effect of chromosome length (Table 1). The range of cytogenetic stocks analyzed (centric translocations, deletion lines, additions, and telosomic lines) made it possible to analyze spatial positioning using independent tests involving various configuration of 1R or its arms, with varying chromosome lengths, chromosome arm lengths and arm ratios (test 1 and 2 for positioning of 1RS and 1RL, respectively), six centric translocations lines (test 3), two addition lines of wheat chromosomes to tetraploid rye (test 4), two whole-chromosome additions of barley chromosomes to wheat (3H, 6H) (test 5), and four telosomic additions of barley chromosome arms to wheat (test 6). Similarly, the arms of the longest wheat chromosome, 3B, present in rye, are more peripherally located than the arms of 5B, a shorter wheat chromosome. Barley introgressions in wheat show a similar pattern: chromosome arms of the longer chromosome 3H occupy more peripheral positions than those of the shorter chromosome 6H (Table 1).

Test 1: Deletion in 1RS in _del_1RS.1RL resulted in a much more central position of this arm than in a normal 1R. Interestingly, separating 1RS from the long arm in t1RS changes the positioning of this arm to the most peripheral among all the 1RS arms analyzed in this study. The MA-CN distances are different for the 1RS arm in different configurations (1R, _del_1RS.1RL, 1RS._del_1RL, and t1RS). These configurations formed two homogeneous groups: one containing 1R(1A) and t1RS introgressions with significantly higher MA-CN than the second group, containing all the remaining 1RS introgressions (1RS._del_1RL, _del_1RS.1RL). There is a strong and significant positive correlation between MA-CN and the arm length (*r_s_* = 1.00, *p* << 0.001), a weak positive correlation of MA-CN with the chromosome length (*r_s_* = 0.50, *p* = 0.67) and a negative correlation with the arm ratio (*r_s_* = −0.50, *p* = 0.66). As telocentric t1RS only comprises a single chromosome arm, it was excluded from the analysis of correlations between MA-CN and chromosome length and between MA-CN and the arm ratio.

Test 2: Variation in MA-CN among introgression lines was also observed for the 1RL arm. The 1RL arm reduced in length by ca. 30% by a deletion is located in a more central position in the nucleus relative to the complete arm. The short arm appeared to play a role in positioning of the long arm: 1RL displayed the most peripheral location when present in 1R, followed by 1RS._del_1RL and _del_1RS.1RL (Table 1). Correlations between MA-CN and chromosome length (r_s_ = 1.00, *p* << 0.001), chromosome arm length (*r_s_* = 1.00, *p* << 0.001), and arm ratio (*r_s_* = 0.50, *p* = 0.67) are positive and highly significant.

Test 3: Centric translocations significantly differ in the MA-CN distances, and they form two homogeneous groups: one containing 1RS.1DL and 5RS.5BL, with significantly shorter MA-CN than the second group containing all the remaining centric translocations (Table 1). There are weak to strong positive correlations between MA-CN and chromosome length (*r_s_* = 0.71, *p* = 0.11), the arm length (*r_s_* = 0.83, *p* = 0.04), and arm ratio (*r_s_* = 0.26, *p* = 0.62).

Test 4: 3B and 5B introgressions into tetraploid rye do not differ with respect to the MA-CN distance for either the short or long arms (Table 1). There is a clear tendency of MA-CN to increase with the chromosome length and chromosome arm length, and decrease with the arm ratio for the short arms. For the long arms, MA-CN increased with chromosome length and decreased with both the arm length and arm ratio (not tested due to *n* = 2).

Test 5: 3H, 6H introgressions in wheat significantly differ with respect to the MA-CN distance for both the short and long arms (Table 1). There is a clear tendency for MA-CN to increase with the chromosome length, chromosome arm length, and arm ratio for both short and long arms (not tested due to *n* = 2).

Test 6: 3HL, 3HS, 6HL, 6HS introgressions in wheat did not differ with respect to the MA-CN distance (Table 1). There is a weak positive correlation between MA-CN and the chromosome arm length (*r_s_* = 0.40, *p* = 0.60).

All six tests support the hypothesis that chromosome length and chromosome arm length affect spatial positioning of a chromosome arm in a three-dimensional (3D) nucleus. Longer chromosomes and longer chromosome arms tend to be more peripherally located, while shorter chromosomes and chromosome arms are preferentially located in the interior of the nucleus. Regardless of the positioning of the arm in the nuclear volume, all of the chromosomes and arms still display the Rabl’s organization.

### 2.3. The Effect of the Cell Cycle Stages and Tissue-Specificity

**Figure 3:**
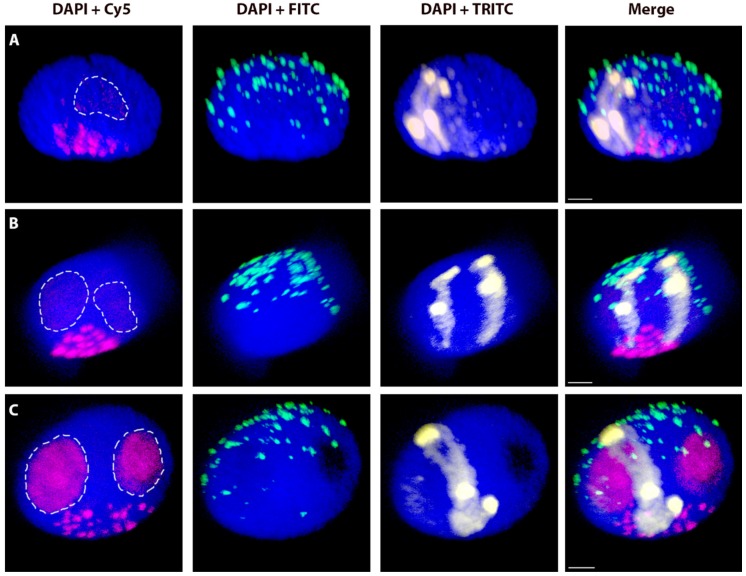
Wheat nuclei with introgressed pair of rye _del_1RS.1RL chromosomes in different cell cycle stages: G_1_ phase (**A**), S phase (**B**) and G_2_ phase (**C**). Total genomic DNA of rye was labelled with TRITC using Nick translation (yellow), centromeres of both wheat and rye chromosomes were visualized using oligonucleotide probe (magenta), and the telomere-specific sequence was PCR-labelled with FITC (green). Nuclear DNA was counterstained with DAPI (blue). Nucleoli are indicated by white dashed lines. Scale bar = 3 µm. Note more central positioning of rye chromosomes in the progression of cell cycle and no resolution of sister chromatids in G_2_.

To evaluate the possible changes of nuclear characteristics and chromosome positions in various stages of the cell cycle, the 3D-FISH experiments were repeated on the G_2_ and S-phases nuclei that were isolated from root tips of four selected wheat-rye introgression lines (Table 3). Morphologically, the nuclei in G_2_ and S were similar to those in G_1_ (Figure 3) and so the same shape criteria were used to select nuclei for analyses and the same parameters were measured. As in G_1_, introgressed chromosomes or chromosome arms are arranged in Rabl’s configuration with their centromeres and telomeres being located on the opposite poles of the nuclei. The fluorescent signal from the genomic probe did not allow for us to distinguish the two chromatids during or after DNA replication in S- or G_2_-phase nuclei, respectively.

In total, 85 nuclei in the S phase and 90 nuclei in G_2_ were analyzed. Their volumes ranged from 1252 to 3236 µm^3^ (average 2076 µm^3^) and from 1061 to 4313 µm^3^ (average 2456 µm^3^) for S and G_2_ phases, respectively. The lengths of individual rye chromosome arms in the S-phase ranged from 1.4 to 11.4 µm, and from 2.2 to 9.9 µm in S and G_2_, respectively. Relative to the values that were observed in the G_1_ nuclei (Table 1), introgressed rye chromosome arms appear to be shorter with the cell cycle progression (they are the longest in G_1_ and the shortest in G_2_), while the volume of the nucleus increases (it is the smallest in G_1_ and the largest in G_2_) (Table 3). Introgressed chromosome arms usually display the most peripheral position in G_1_ and a more central position in G_2_ and S nuclei. Interestingly, the shift to more central positioning of 1RS reduced in length by a deletion in _del_1RS.1RL that is evident in G_1_, is not observed in S and G_2_.

**Figure 4:**
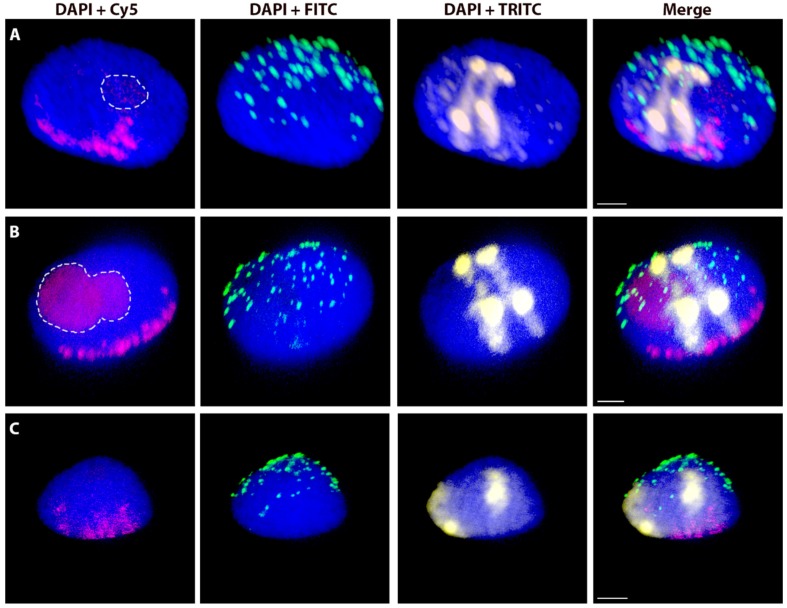
Wheat nuclei with a pair of introgressed rye _del_1RS.1RL chromosomes in various tissues. A nucleus from root tips (**A**), embryos (**B**) and leaf tissue (**C**). Total genomic DNA of rye was labelled with TRITC using Nick translation (yellow), centromeres of both wheat and rye chromosomes were visualized using the oligonucleotide probe (magenta), and telomere-specific sequence was PCR-labelled with FITC (green). Nuclear DNA was counterstained with DAPI (blue). Nucleoli are indicated by white dashed lines. Scale bar 3 µm.

The same 3D-FISH experiments were repeated again, on G_1_ nuclei from embryonic and leaf cells of two selected wheat-rye introgressions to assess the possible variability of nuclear characteristics and chromosome positions in various plant tissues (Table 4). The morphology of nuclei that were isolated from both tissues, as well as the general organization of chromosomes in the Rabl’s configuration, appeared to be similar to the nuclei that were isolated from root tips (Figure 4).

In total, 44 nuclei from embryonic cells and 41 nuclei from leaf mesophyll were analyzed. The nuclear volumes ranged from 938 to 2064 µm^3^ (average 1300 µm^3^) in embryonic cells and from 363 to 927 µm^3^ (average 651.5 µm^3^) in the leaf cells. The lengths of individual rye chromosome arms ranged from 2.8 to 13.7 µm and from 2 to 19.5 µm in nuclei from embryos and from leaves, respectively. When compared to the values that were obtained from the G_1_ nuclei from root tips (Table 1), the largest nuclear volume was found in root tips, while the smallest in nuclei from leaf cells. Despite this, the introgressed chromosome arms appeared to be the longest in the root tip nuclei and in embryonic cells, and the shortest in the leaf cell nuclei. In a majority of cases, the distances between introgressed homologous chromosome arms as well as their distances from the centre of the nucleus appear to be the longest in leaf cell nuclei and the shortest in root tip nuclei.

### 2.4. Spatial Separation vs. Association of Alien Chromosome (Chromosome Arm) Homologues

**Figure A4:**
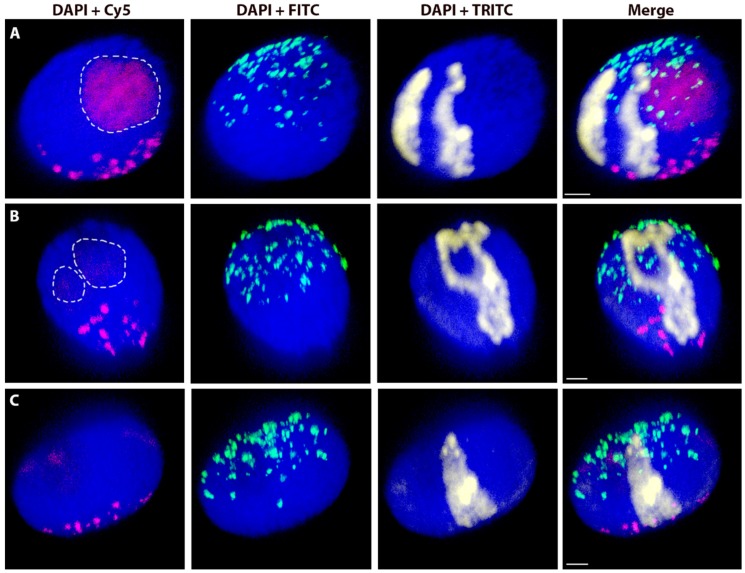
Wheat nuclei with a pair of homologous rye chromosomes _del_1RS.1RL in wheat background. (**A**) Complete separation (**A**), parttaTial association (**B**) and complete association (**C**) of rye homologues. Total genomic DNA of rye was labelled with TRITC using Nick translation (yellow), centromeres of both rye and wheat chromosomes were visualized using oligonucleotide probe (magenta), and telomere-specific sequence was PCR-labelled with FITC (green). Nuclear DNA was counterstained with DAPI (blue). Nucleoli are indicated by white dashed lines. Scale bar 3 µm.

Visual screening revealed that the territories of pairs of introgressed homologous chromosomes and chromosome arms adopt various types of arrangements (Figure A4). The most frequently observed arrangement across all lines in the G_1_ nuclei from root tips (83%) is the complete separation of homologues (Table 5). Complete association along entire homologous arms is evident in only 3.7% of nuclei; 13.3% of nuclei display their partial association. Rye homologues introgressed into the wheat background are completely separated in 82.9% of G_1_ nuclei. Partial chromosome association is observed in 14.6% of nuclei, and the complete association of rye homologues was observed in 2.5% of nuclei. Interestingly, in the rye background, not a single nucleus with fully associated wheat homologues was observed; only 5% of such nuclei show partial association, while wheat homologues are completely separated in the remaining nuclei (95%).

The frequencies for barley chromosomes or chromosome arms introgressed into wheat are similar to those of rye introgressions. The complete separation of barley homologues is observed in 79.4% of nuclei, while the partial and complete association is displayed by 12.7% and 7.9% of nuclei, respectively.

3D-FISH of embryonic G_1_ nuclei from two selected wheat-rye introgression lines shows the complete separation of introgressed homologues in 79.5% of nuclei (Table 5). While 11.4% of embryonic nuclei displayed partial association of rye homologues, complete association is found in 9.1% of nuclei. Similarly, 80.4% of the G_1_ nuclei from leaves showed a complete separation of the introgressed chromosomes or chromosome arms. The frequency of both complete and partial associations of rye homologues is the same (9.8% of nuclei). Higher frequencies of complete associations of the introgressed chromosomes or chromosome arms both in leaf and embryonic nuclei, as compared to that obtained from nuclei of root tips were observed.

Rye chromosomes or chromosome arms are completely separated in 80% of G_2_ nuclei of four selected wheat-rye introgression lines (Table 5). In 13.3% of the G_2_ nuclei, the rye homologues show partial association and only 6.6% of G_2_ nuclei showed complete association of rye homologues. In a majority of the S-phase nuclei (87%), rye chromosomes or chromosome arms are completely separated. Partial association is observed in 10.6% of the S-phase nuclei and only 2.4% of S-phase nuclei have completely associated rye chromosomes or chromosome arms. Thus, there are only slight differences when compared to the frequencies that were obtained from the G_1_ nuclei.

## 3. Discussion

The organization of DNA in a nucleus affects many fundamental biological functions, such as replication, gene expression, and chromosome segregation [37]. However, the mechanisms governing spatial organization of CTs in plant cell nuclei, and even chromatin organization itself, are still largely unknown. Large chromosomes in species with large genomes, such as wheat, barley, and rye, assume the Rabl’s configuration, while species with small chromosomes and small genomes seem to display different organizations, such as the Rosette observed in *Arabidopsis* [17,29,38,39,40]. However, genome size *per se* may not be the critical factor in this regard. The current view may be biased toward the species with large genomes, because they are favored as objects of cytogenetic research. Observations in *Brachypodium* have shown that species with small genomes and chromosomes might display the Rabl’s configuration: *B. distachyon* (L.) P. Beauv. with a small genome, shows the Rabl’s configuration, while *B. stacei* Catalan, Joch.Muell., L.A.J.Mur & T.Langdon and *B. hybridum* Catalán, Joch. Müll., Hasterok & Jenkins with larger genomes appear to have a more random organization of chromosome territories [41]. In the present study, Rabl’s orientation of rye chromosome arms was observed in all nuclei. The centromeres were generally more tightly clustered than the telomeres, which is probably a relic of the last anaphase movement. Similarly, Idziak et al. [41] showed that telomeres occupy a larger nuclear space as compared to clusters of centromeric sequences in *Brachypodium*.

The association of homologues or their parts during interphase may facilitate DNA repair via homologous recombination and enable the contacts of genes with their regulatory elements to initiate transcription [42]. However, this study shows that such an association of introgressed homologues is infrequent (~17%). This agrees with a theoretical frequency of random positioning for 42 chromosomes (11.95%) in wheat and with the results of Berr and Schubert [19]. They also found a slightly higher frequency of associations of homologues in the spherical nuclei of various tissues of *Arabidopsis thaliana* than predicted by the SCD (spherical (1Mb) chromatin domain) model of Cremer et al. [8]. Similarly, Mascher et al. [36] identified contacts among chromosomes in barley while using the Hi-C method and found that contacts between homologues only accounted for 13.1–20.9% (16.5%, on average) of all contacts. The spatial association of homologues has been rarely observed in other disomic chromosome introgression lines, using in situ hybridization of squashed tissue samples [43,44]. On the other hand, preferential association of homologues, exceeding random positioning, was observed in the nuclei of *Brachypodium distachyon* and *Drosophila melanogaster* [45,46]. Interestingly, Baroux et al. [47] found the preferential association of one paternal and one maternal homologue in endosperm nuclei of *Arabidopsis* (a triploid tissue), leaving one maternal homologue non-associated. In our case, variation in association with homologues among different tissues was also marginal: complete separation ranged from 80% in embryonic and leaf nuclei to 88% in root cells. Similarly, Berr and Schubert [19] found only slight variation in the degree of separation of homologues between spherical nuclei of cotyledon, shoot, stem, and root tissues.

Hiraoka et al. [48] suggested that factors beyond the tissue type, such as the stage of the cell cycle and development, might also affect the spatial alignment of homologues. However, there are no indications that the CT movement occurs during interphase [49,50]. The observations presented here fully support that notion: there were only minor differences among nuclei of four genotypes (rye introgressions in wheat) in different phases of the cell cycle, and in different tissues. Similarly, chromosome arm length was proposed to affect homologue associations, with shorter arms being less likely to be associated than the longer arms [45]. No such correlation was observed in this study. Longer chromosome arms did not associate any more frequently than the shorter ones. Only slight differences were observed among the various stocks tested (wheat chromosomes in rye, barley chromosomes or chromosome arms in wheat, and rye chromosomes or chromosome arms in wheat). This indicates that the organization of chromatin in the 3D space of a nucleus is highly predetermined, at least in the genomes that are involved in this study (wheat, barley, and rye).

On the other hand, considerable variability in the association/separation of homologues among translocation lines may reflect the dynamics of chromatin organization. Variability and movement of chromatin during interphase were observed in several organisms [51]. Chromosomes probably do not actively change their positions, but rather display limited, diffusive movements [52,53], with interstitial chromosome segments generally showing more movement than the centromeres [54]. However, genome organization seems to be flexible enough to allow for the contacts of homologues necessary for DNA repair and initiation of transcription. On the other hand, the general immobility of chromatin plays a key role in the maintenance of genomic stability [55].

It has been reported that cellular differentiation has no major impact on the nuclear organization in various tissues in *A. thaliana* and barley [19,56]. In this study, in the positioning of rye chromosomes during the cell cycle were observed, apart from minor variation in the separation of homologues in nuclei at different phases of the cell cycle, changes. Not surprisingly, the nuclear volume increased during transition through the cell cycle, while the introgressed rye chromosome arms (or chromosomes) became shorter. Interestingly, as they became shorter, they tended to assume more central positions in the nucleus, but were still within Rabl’s orientation. This phenomenon was common to all four genotypes tested.

Six independent tests that involved all three types of genotypes (wheat introgression in rye, rye in wheat, and barley in wheat) that were conducted in this study indicated that shorter chromosome arms are more likely to be positioned in the nuclear interior, whereas longer ones are often located at the nuclear periphery. Apart from the chromosome arm length, the total chromosome length also appears to play a role. The same rye chromosome arm 1RS behaved differently when translocated to two different wheat chromosome arms, 1BL and 1DL. The same identical 1RS is more peripheral in 1RS than in 1RS.1DL. Wheat chromosome arm 1BL is considerably longer than 1DL, hence, the translocation chromosome 1RS.1BL is also longer. This difference in positioning of the same chromosome arm present in two translocations of different lengths implies that the position of a chromosome arm in the volume of the nucleus is a function of the chromosome size, and that the length of one arm may affect the position of the entire chromosome. This is further supported by the positioning of the same arms reduced in length by deletions. It appears that the positioning of a chromosome/chromosome arm in the volume of the nucleus, at least in the case of rye introgressions in wheat, is not chromosome-specific (that is, dependent on its gene content), but rather a function of chromosome length. A change in the overall length of a chromosome, such as by translocation to shorter or longer wheat arm, or reduction by deletion, affects the position of the entire chromosome, and not only of the directly affected arm (Table 1). While the general rules of chromosome positioning in the nuclei of plants and animals appear to be different, Sun et al. [3] and Bolzer et al. [57] reported that smaller human chromosomes were internally positioned and larger chromosomes were close to the nuclear envelope. It is not clear whether or not the length of the chromosome arm itself determines its position. In species with non-Rabl’s configuration, it appears that gene density is the critical factor. Gene-dense chromosomes are predominantly positioned close to the nuclear centre while chromosomes with lower gene densities are located towards the nuclear periphery [1,4]. Here, the gene content of all group-1 chromosomes (1A, 1B, 1D, and 1R) is essentially the same, so gene density is a function of the chromosome/chromosome arm length: translocation of the same 1RS to two different wheat arms, both essentially with the same gene content but different length, changes gene density of the entire chromosome. On the other hand, deletions of the gene-poor proximal regions of arms also elevate the gene density of the entire chromosome.

According to Bauer et al. [58], rye chromosome 5R has the highest gene density (3.46 genes per 1Mb), followed by 1R (3.18 genes per 1Mb); gene density in 2R is relatively low (2.83 genes per 1Mb). The physical lengths of the three chromosomes follows the opposite pattern, with 5R being the shortest and 2R the longest in the genome [31]. In this study, 5RS was preferentially located close to the nuclear centre, whereas 2RS more frequently occupied the peripheral positions. However, if there is a general correlation between chromosome length and gene density in rye, there appears no clear way to separate the two factors on chromosome positioning. Different behavior of the same rye arm in two different translocations, 1RS.1DL and 1RS.1BL, each producing a chromosome of different total length, might provide a reasonable approach. There are many clear length differences among homoeologues in wheat. Thus, the same rye chromosome arm translocation to the shortest wheat homoeologue (usually in the D genome) would produce a chromosome of a different total length than translocation to a B-genome homoeologue (usually the largest in the wheat genome). On the other hand, it has not been clarified whether these position effects are of general validity. Random organization of the chromosomes appears to be the case in *Arabidopsis* with the exception of chromosomes 2 and 4. These two chromosomes carry NORs, and since NORs in most cases organize a single nucleolus, these two pairs of chromosomes are physically located more closely to one another than the rest of the genome [16].

Intermingling of the parental genomes in interspecific hybrids with equal proportion of genomes, such as in F1 hybrids of parents with the same numbers of chromosomes (of the same or similar length), is another issue. The pioneering work using sectioned interphase nuclei revealed that genomes of *Hordeum vulgare* and *Secale africanum* Staph. are spatially separated in the *H. vulgare × S. africanum* hybrid throughout the cell cycle [59,60]. Moreover, the genome that originates from *H. vulgare* L. tended to be located in a single cluster and positioned centrally in the nucleus. Similarly frequent separation of *Lolium* L. and *Festuca* L. chromatin in interphase nuclei was observed in their F1 hybrids (Kopecký et al., unpublished). On the other hand, not a single nucleus was observed in this study with clear spatial separation of the genomes of wheat and rye (as two hemispheres with the least possible contacts between them), neither in tetraploid nor in hexaploid triticale (Figure 2). Thus, the spatial separation of parental genomes in allopolyploids during the cell cycle is still an open question and more experiments have to be conducted to resolve this issue.

To conclude, this study shows that the chromosome domains of introgressed chromosomes or chromosome arms are highly stable among the tissue types and during the cell cycle phases. Chromosomes uniformly display the Rabl’s configuration and homologues are spatially separated, as predicted by the theoretical model. All of the experiments and tests support the hypothesis that shorter chromosomes and chromosome arms are more centrally located in the 3D nucleus, while longer ones are peripherally positioned, close to the nuclear envelope. On the other hand, it appears that the arm ratio itself does not play a role in the positioning of the chromosome and its arms.

## 4. Materials and Methods

### 4.1. Plant Material

The plant material consists of a set of lines of common wheat (*Triticum aestivum* L. ssp. *aestivum*, 2*n* = 6× = 42) cv. ‘Pavon 76’ with introgressions of rye chromosomes or chromosome arms: disomic substitution of rye chromosome 1R for wheat chromosome 1A [1R(1A)], ditelosomic addition line 1RS, a deletion line _del_1RS.1RL(1A), where ca. proximal 36% of the short arm is missing, a deletion line 1RS._del_1RL(1A) where proximal ca. 30% of the long arm is missing, and homozygotes for centric wheat-rye chromosome translocations 1RS.1BL, 1RS.1DL, 1AS.1RL, 2RS.2BL, 2BS.2RL, and 5RS.5BL. The telosomic line and all centric translocation lines were created by the centric misdivision of complete normal chromosomes of rye and their wheat homoeologues; the deletion chromosomes were identified during selection of centric translocations [61].

Further, a set of alien chromosome addition lines carrying barley (*Hordeum vulgare* L.) cv. ‘Manas’ chromosomes or chromosome arms in a common wheat (original cross with cv. ‘Asakaze’, backcrossed to cv. ‘Chinese Spring’) background (3H, 3HL, 3HS, 6H, 6HL, 6HS) and two lines carrying disomic wheat chromosomes 3B and 5B in a tetraploid rye background was used. Additionally, tetraploid triticale with the genomic constitution AARR and hexaploid triticale cv. ‘Rhino’ with genomic constitution AABBRR were also involved in the study. All of the materials with rye chromosomes (wheat-rye addition and translocations lines, rye-wheat introgression lines and triticales) were provided by Dr. A.J. Lukaszewski, University of California, Riverside, CA, USA.

Note on terminology: this manuscript uses the original terminology of Bridges (1917) for chromosome aberrations, where “deficiency” is a loss of a terminal chromosome segment and “deletion” indicates a loss of an intercalary segment.

### 4.2. Isolation of Nuclei by Flow Cytometry Sorting

The isolation of nuclei was performed according to [62]. Nuclei in G_1_, G_2_, or S phase of the cell cycle were identified and sorted while using a FACSAria II SORP flow cytometer (BD Biosciences, San Jose, CA, USA). About 50,000 nuclei at G_1_, G_2_, or S phase were obtained for each sample.

Dry seeds were put into water over night to obtain embryonic cells, and after the removal of seed coats they were fixed, as described above. For leaf cells, leaves from mature plants were collected into a tube and fixed in the same way. Seeds and leaves were cut in the meiocyte buffer A to very small pieces by a razor blade. The obtained mixture was filtered through 20 µm nylon mesh into a 5 mL polystyrene tube and processed, as described above.

### 4.3. Probe Preparation and 3D-FISH

Total genomic DNA of *Secale cereale* L. and *Hordeum vulgare* was labelled with Texas Red or TRITC while using a Nick Translation Kit (Roche Applied Science, Penzberg, Germany) according to manufacturer’s instructions, and applied as a probe. A centromeric probe was prepared by PCR using centromere-specific primers [63] and digoxigenin-11-dUTP (Roche Applied Science). The probe was detected with anti-digoxigenin-fluorescein (Roche Applied Science). In some experiments, a combination of telomeric and centromeric probes was used. For the wheat and rye centromeres, an oligonucleotide probe that was based on the sequence of clone pHind258 [63] and directly labelled with Cy5 was used. The telomeric probe was prepared while using PCR and FITC-directly labelled nucleotides. Total genomic DNA of wheat was sheared to 200–500 bp fragments by boiling and used as blocking DNA at a ratio of 1:150 (probe/blocking DNA). To visualize wheat chromosomes in rye background, total genomic DNA of *T. durum* Desf. was labelled with TRITC and then combined with rye blocking DNA (both prepared as described above). For experiments with triticale, rye genomic DNA was labelled with FITC and genomic DNA from *T. durum* was labelled with TRITC (both prepared using a Nick Translation Kit). 3D-FISH experiments were performed, as described in [62].

### 4.4. Image Acquisition and Analysis

Selected nuclei were optically sectioned while using an inverted laser spinning disk microscope (Axio Observer Z1, Zeiss, Oberkochen, Germany) and ZEN Blue 2012 software; an inverted motorized microscope Olympus IX81 equipped with a Fluoview FV1000 confocal system (Olympus, Tokyo, Japan) and FV10-ASW software; and Axio Imager.Z2 microscope (Zeiss) equipped with confocal Andor DSD2 System and iQ3.6 software (Andor, Belfast, United Kingdom of Great Britain).

For each nucleus, 80–120 optical sections in 160–200 nm steps were taken and then merged into a 3D model. Subsequent analyses were performed while using Imaris 9.2 software (Bitplane, Oxford Instruments, Zurich, Switzerland). Imaris applications ‘Contour Surface’, ‘Spot Detection’ and ‘3D Measurement’ were used for the manual analysis of each nucleus. The volume and the centre of the nucleus (CN) were determined from the rendering of primary intensity of DAPI staining while using the function ‘Surfaces’. The lengths of introgressed chromosome arms were measured using the ‘Polygon’ function. The ‘Spot’ function was used to mark the positions of centromeres (C), telomeres (T), and the mid-points of introgressed chromosome arms (MA). Distances between the centromeres of introgressed homologues (C-C), between their telomeres (T-T) and between their mid-points of arms (MA1-MA2) were measured while using the ‘Line’ function. For more precise assessment of the distance between the centre of the nucleus and the chromosome arm (CN-MA), distances between three points of the arm (at 25%, 50%, and 75% of the arm length) and the centre of the nucleus for each chromosome arm were measured. Averages of these 3 values were used in subsequent analyses. The nuclear volume of triticale nuclei was calculated as the sum of volumes of the rye and wheat chromatin, both being measured using the ‘Surfaces’ function. ‘Display Adjustment’ was used to adjust the channel contrast and, thus, to improve the visualization of all analyzed objects. Between 20 and 40 nuclei were analyzed per genotype.

### 4.5. Association vs. Separation of Rye Homologous Chromosome Arms in the Wheat Nucleus

The nuclei were visually screened in Imaris to estimate the frequency of association (manifesting itself as physical connection) or separation of targeted chromosomes or chromosome arms, and each nucleus was assigned to one of the categories: 1, complete separation, where no visible connection of the homologous arms was observed; 2, partial association, where a connection in at least a short segment of the chromosome arms was visible; and, 3, complete association, where both homologous chromosomes or chromosome arms were associated along their entire lengths.

### 4.6. Statistical Data Analysis

The distance between the mid-point of introgressed arms and the centre of nucleus (MA-CN) was used as the main determinant of spatial positioning of introgressed chromosome/chromosome arms in a 3D nucleus. The MA-CN values were compared between the translocation lines while using general linear models (LM) and individual comparison tests were done using Bonferroni adjusted probability level after a significant F test. A similar statistical procedure was used to compare the nuclear volume and arm length. The Spearman correlation coefficient was used to evaluate the relationship between chromosome arm length, chromosome length, arm ratio, and the MA-CN. The MA-CN distances were standardized by nuclear volume as a dependent variable to avoid potential bias caused by nuclear volume variation within and between translocation lines. For statistical evaluation, estimated values of arm ratio, chromosome, and chromosome arm length have been calculated from karyotypes of Schlegel et al. (1987) and Naranjo (2018) for rye, Gill et al. (1991), Paux et al. (2008) and Salina et al. (2018) for wheat, and Mascher et al. (2017) for barley and genome size estimations (Doležel et al., 1998). All statistical analyses were done in NCSS 9 (NCSS 9 Statistical Software; NCSS, LLC; Kaysville, Utah, USA, ncss.com/software/ncss).

## Figures and Tables

**Table 1:** Morphometric characteristics of introgressed chromosome arms in wheat-rye and wheat-barley introgression lines.

Introgression	Introgressed Chromosome Arm Length^3^ (Mb)	Chromosome Length (Mb)	Arm Ratio	Nuclear Volume (µm^3^)	Arm Length (µm)	C-C (µm)	MA1-MA2 (µm)	T-T (µm)	CN-MA (µm)
1AS.1RL	626	902	2.27	1545 ± 228	7.73 ± 1.48	4.01 ± 1.98	5.11 ± 2.28	4.88 ± 1.71	3.55 ± 0.84^a^
1RS.1BL	423	959	1.27	1501 ± 362	8.21 ± 1.69	3.49 ± 1.50	4.42 ± 1.64	4.18 ± 1.66	3.57 ± 1.01^a^
1RS.1DL	423	804	0.90	1998 ± 285	6.86 ± 1.31	2.83 ± 1.15	3.55 ± 1.63	3.71 ± 1.74	2.82 ± 0.78^b^
2RS.2BL	595	1102	0.85	1597 ± 490	8.51 ± 2.58	3.38 ± 1.70	4.38 ± 2.04	4.09 ± 1.95	3.51 ± 1.22^a^
2BS.2RL	693	1116	1.64	1510 ± 357	8.96 ± 1.62	4.05 ± 1.80	4.99 ± 2.13	4.99 ± 2.25	3.76 ± 0.97^a^
5RS.5BL	346	928	1.68	1656 ± 329	6.95 ± 1.54	3.06 ± 1.29	3.68 ± 1.47	3.97 ± 1.48	2.99 ± 0.91^b^
Test statistics	F (dfg, dfe)*^p^*^-Value^	-	-	-	-	-	-	-	13.55 (5, 195.6)***
_del_1RS.1RL	S^1^: 271	897	2.31	1986 ± 390	4.04 ± 1.03	2.88 ± 1.37	3.52 ± 1.50	3.86 ± 1.79	2.82 ± 0.98^a^
	L: 626				7.56 ± 1.67		3.87 ± 1.74	3.56 ± 1.87	3.13 ± 0.87^A^
1RS._del_1RL	S: 423	861	1.04	2006 ± 500	6.18 ± 1.58	3.57 ± 1.69	4.19 ± 1.88	4.20 ± 1.92	3.05 ± 1.00^a^
	L^2^: 438				5.21 ± 1.41		4.06 ± 1.83	4.12 ± 1.53	3.09 ± 1.00^A^
1R(1A)	S: 423	1049	1.48	1196 ± 143	8.58 ± 1.79	4.09 ± 2.41	5.45 ± 2.27	4.99 ± 2.32	4.12 ± 1.27^b^
	L: 626				9.88 ± 1.87		5.22 ± 2.76	4.23 ± 2.47	4.09 ± 1.24^B^
t1RS	423	423	-	1190 ± 109	8.84 ± 1.77	4.15 ± 1.86	4.94 ± 2.26	4.67 ± 2.09	4.21 ± 1.19^b^
Test statistics	F (dfg, dfe)*^p^*^-Value^	-	-	-	-	-	-	-	S: 18.76 (3, 93.9.)*** L: 10.57 (2, 82.5)***
3B	S: 433	995	1.30	1535 ± 315	5.63 ± 1.47	4.30 ± 1.52	5.39 ± 1.7	5.35 ± 2.02	3.83 ± 0.98
	L: 562				6.91 ± 1.81		4.96 ± 1.79	4.22 ± 1.93	3.76 ± 1.03
5B	S: 290	870	2.00	1492 ± 383	4.30 ± 1.67	4.48 ± 1.93	4.96 ± 1.86	4.82 ± 1.62	3.65 ± 1.24
	L: 580				6.96 ± 1.94		5.13 ± 1.86	3.61 ± 1.77	3.60 ± 1.07
Test statistics	F (dfg, dfe)*^p^*^-Value^	-	-	-	-	-	-	-	S: 0.54 (1, 74.2)^n.s.^ L: 0.46 (1, 77.9)^n.s.^
3H	S: 294	700	1.38	1316 ± 328	5.74 ± 1.48	3.7 ± 2.2	4.54 ± 2.26	5.25 ± 2.46	3.98 ± 1.43
	L: 406				6.96 ± 1.94		3.95 ± 1.98	4.36 ± 1.88	3.70 ± 1.25
6H	S: 261	583	1.23	1848 ± 352	4.13 ± 1.78 (3.46 ± 1.08)	3.77 ± 1.59	4.22 ± 1.71	4.99 ± 1.76	3.18 ± 1.00
	L: 322				5.68 ± 1.69		4.29 ± 2.08	4.71 ± 1.96	3.05 ± 1.08
Test statistics	F (dfg, dfe)*^p-^*^Value^	-	-	-	-	-	-	-	S: 8.30 (1, 69.9)** L: 6.13 (1, 76.4)*
3HL	406	406	-	1419 ± 358	8.19 ± 1.91	2.88 ± 1.49	3.81 ± 2	3.93 ± 2.64	3.65 ± 1.12
3HS	294	294	-	2182 ± 646	4.14 ± 0.97	2.73 ± 1.66	3.08 ± 1.93	3.33 ± 1.99	3.19 ± 1.10
6HL	322	322	-	1695 ± 307	5.46 ± 1.33	2.9 ± 2.06	3.98 ± 2.83	3.97 ± 2.97	3.34 ± 1.29
6HS	261	261	-	1868 ± 609	6.65 ± 1.79 (4.23 ± 1.06)	3.15 ± 1.76	4.26 ± 2.24	4.59 ± 2.45	3.35 ± 1.16
Test statistics	F (dfg, dfe)*^p^*^-Value^	-	-	-	-	-	-	-	1.37 (3, 92.7)^n.s.^

The values (mean ± SD) of arm length, distances between centromeres (C-C), between mid-points of the arms (MA1-MA2), between telomeres (T-T), and arm mid-points to the centre of nucleus (MA-CN) were standardized according to the volume of the nucleus (absolute distance in µm/nuclear volume × 1000). For 6H and 6HS, the arm length without nucleolus is given in brackets. Differences in mean values of centre of the nucleus and the chromosome arm (CN-MA) among introgression lines were tested by general linear models using the F-test. Individual comparison tests were done using the Bonferroni adjusted probability level. Significantly different means of respective characteristic among introgression lines are coded by different letters column-wise and separately for each introgression group and short/long arm, if available (*p* Value coding: *** *p* ≤ 0.001, ** 0.001 < *p* ≤ 0.01, * 0.01 < *p* ≤ 0.05, n.s., not significant with *p* > 0.05). ^1^ deletion of proximal about 36% of 1RS; ^2^ deletion of proximal about 30% of 1RL; ^3^ estimated values of chromosome and chromosome arm length and arm ratios were calculated from rye karyotypes of Schlegel et al. [30] and Naranjo [31], from Gill et al. [32], Paux et al. [33] and Salina et al. [34] for wheat, and from Mascher et al. [35] for barley and genome size estimations [36].

**Table 2:** Characteristics of G_1_ nuclei of tetraploid and hexaploid triticale (mean ± SD).

Triticale	Number of Nuclei	Nuclear Volume (µm^3^)	Rye Chromatin (%)	Wheat Chromatin (%)
4×	22	993 ± 260	53.6 ± 4.7	46.4 ± 4.7
6×	22	1412 ± 274	37.7 ± 10.0	62.3 ± 10.1

**Table 3:** Morphometric characteristics of rye chromosome arms in G_2_ and S-phase nuclei of selected wheat-rye introgression lines. The values (mean ± SD) of arm lengths, distances between centromere to centromere (C-C), the mid-point of arm to the mid-point of arm (MA1-MA2), telomere to telomere (T-T), and mid-point of arms to centre of nucleus (MA-CN) were standardized according to the volume of the nucleus (absolute distance in µm/nuclear volume × 1000). Differences in mean values of selected characteristics among the cell cycle stages within each introgression were tested by general linear models using the F-test. Individual tests were done using the Bonferroni adjusted probability level. Significantly different means of respective characteristic among stages are coded by different letters column-wise and separately for each introgression and short/long arm, if available (*p* value coding: *** *p* ≤ 0.001, ** 0.001 < *p* ≤ 0.01, * 0.01 < *p* ≤ 0.05, n.s., not significant with *p* > 0.05).

Introgression	Cell Cycle Stage	Nuclear Volume (µm^3^)	Arm Length (µm)	C-C (µm)	MA1-MA2 (µm)	T-T (µm)	MA-CN (µm)
1AS.1RL	G_1_	1545 ± 228^a^	7.73 ± 1.48^a^	4.01 ± 1.98	5.11 ± 2.28	4.88 ± 1.71	3.55 ± 0.84^a^
	S	1791 ± 400^b^	7.50 ± 1.60^a^	3.17 ± 1.24	4.28 ± 1.76	3.73 ± 1.47	3.23 ± 0.95^a^
	G_2_	2562 ± 428^c^	6.13 ± 1.15^b^	2.12 ± 0.99	2.28 ± 0.74	2.63 ± 1.27	2.43 ± 0.57^b^
Test statistics	F (dfg, dfe)*^p^*^-Value^	104.6 (2, 77.2)***	20.8 (2, 85.9)***	-	-	-	33.3 (2, 83.2)***
_del_1RS.1RL^1^	G_1_	1986 ± 390^a^	S: 4.04 ± 1.03^a^	2.88 ± 1.37	3.52 ± 1.50	3.86 ± 1.79	2.82 ± 0.98
			L: 7.56 ± 1.67^A^		3.87 ± 1.74	3.56 ± 1.87	3.13 ± 0.87^A^
	S	2118 ± 367^a^	S: 3.68 ± 0.85^ab^	2.74 ± 1.45	3.45 ± 1.58	3.91 ± 1.61	2.70 ± 0.92
			L: 6.26 ± 1.07^B^		3.71 ± 1.71	3.09 ± 1.13	2.87 ± 0.91^AB^
	G_2_	2308 ± 246^b^	S: 3.38 ± 0.79^b^	2.17 ± 0.91	2.59 ± 1.27	2.94 ± 1.54	2.54 ± 0.72
			L: 6.25 ± 1.12^B^		2.75 ± 1.46	2.15 ± 1.23	2.59 ± 0.67^B^
Test statistics	F (dfg, dfe)*^p^*^-Value^	12.7 (2, 88.1)***	S: 6 (2, 90.5)** L: 12.1 (2, 89.5)***	-	-	-	S: 1.3 (2, 90.0)^n.s.^ L: 5.72 (2, 89.6)**
1R(1A)	G_1_	1196 ± 143^a^	S: 8.58 ± 1.79^a^	4.09 ± 2.41	5.45 ± 2.27	4.99 ± 2.32	4.12 ± 1.27
			L: 9.88 ± 1.87^A^		5.22 ± 2.76	4.23 ± 2.47	4.09 ± 1.24^A^
	S	2147 ± 507^a^	S: 5.88 ± 1.41^a^	2.47 ± 1.08	3.49 ± 1.74	3.31 ± 1.83	2.74 ± 0.80
			L: 6.66 ± 1.59^B^		3.38 ± 1.77	2.79 ± 1.27	2.77 ± 0.85^AB^
	G_2_	2584 ± 450^b^	S: 5.32 ± 1.15^b^	2.48 ± 1.02	3.29 ± 1.48	3.13 ± 1.11	2.33 ± 0.74
			L: 6.03 ± 1.32^B^		3.16 ± 1.37	2.46 ± 1.08	2.36 ± 0.65^B^
Test statistics	F (dfg, dfe)*^p^*^-Value^	12.7 (2, 88.1)***	S: 6.0 (2, 90.5)** L: 12.1 (2, 89.5)***	-	-	-	S: 1.3 (2, 90.0)^n.s.^ L: 5.7 (2, 89.6)**
t1RS	G_1_	1190 ± 109^a^	8.84 ± 1.77^a^	4.15 ± 1.86	4.94 ± 2.26	4.67 ± 2.09	4.21 ± 1.19^a^
	S	2250 ± 367^b^	5.72 ± 1.23^b^	3.38 ± 1.69	3.77 ± 1.67	3.31 ± 1.68	2.59 ± 0.76**^b^**
	G_2_	2371 ± 402^b^	5.64 ± 1.19^b^	2.52 ± 1.3	3.21 ± 1.71	2.96 ± 1.51	2.39 ± 0.86**^b^**
Test statistics	F (dfg, dfe)*^p^*^-Value^	323.7 (2, 65.0)***	57.7 (2, 82.8)***	-	-	-	37.4 (2, 83.2)***

^1^ deletion of about 36% of 1RS arm (proximal part).

**Table 4:** Morphometric characteristics of introgressed chromosome arms in embryonic and leaf nuclei of selected wheat-rye introgression lines. The values (mean ± SD) of the arm length, distances centromere to centromere (C-C), mid-point of arm to the mid-point of an arm (MA1-MA2), telomere to telomere (T-T), and mid-point of arms to the centre of nucleus (MA-CN) were standardized according to the volume of the nucleus (absolute distance in µm/nuclear volume x 1000). Differences in mean values of selected characteristics among tissue types within each introgression were tested by general linear models using the F-test. Individual comparison tests were done using the Bonferroni adjusted probability level. Significantly different means of respective characteristic among tissue types are coded by different letters columnwise and separately for each introgression and short/long arm, if available (*p* Value coding: *** *p* ≤ 0.001, ** 0.001 < *p* ≤ 0.01, * 0.01 < *p* ≤ 0.05, n.s., not significant with *p* > 0.05).

Introgression	Tissue Type	Nuclear Volume (µm^3^)	Arm Length (µm)	C-C (µm)	MA1-MA2 (µm)	T-T (µm)	MA-CN (µm)
1AS.1RL	Root tip	1545 ± 228^a^	7.73 ± 1.48^a^	4.01 ± 1.98	5.11 ± 2.28	4.88 ± 1.71	3.55 ± 0.84^a^
	Embryo	1390 ± 277^b^	8.43 ± 1.90^a^	3.79 ± 2.00	4.65 ± 2.12	5.25 ± 2.59	3.56 ± 1.07^a^
	Leaf	678 ± 73^c^	11.69 ± 2.6^b^	7.67 ± 3.56	6.79 ± 2.95	6.01 ± 2.8	5.58 ± 1.68^b^
Test statistics	F (dfg, dfe)*^p^*^-Value^	428.3 (2, 70.3)***	37.8 (2, 79.9)***	-	-	-	26.6 (2, 78.3)***
_del_1RS.1RL^1^	Root tip	1986 ± 390^a^	S: 4.04 ± 1.03^a^	2.88 ± 1.37	3.52 ± 1.50	3.86 ± 1.79	2.82 ± 0.98^a^
			L: 7.56 ± 1.67^A^		3.87 ± 1.74	3.56 ± 1.87	3.13 ± 0.87^A^
	Embryo	1210 ± 188^b^	S: 4.45 ± 0.97^a^	4.76 ± 2.31	5.13 ± 2.50	5.52 ± 2.35	4.33 ± 1.06^b^
			L: 8.78 ± 2.03^B^		4.85 ± 2.58	5.40 ± 3.23	4.23 ± 1.14^B^
	Leaf	626 ± 131^c^	S: 5.75 ± 1.74^b^	7.10± 3.25	8.73 ± 3.07	9.46 ± 3.01	6.58 ± 2.30^c^
			L: 12.36 ± 2.98^C^		8.48 ± 3.88	7.8± 3.86	6.21 ± 2.44^C^
Test statistics	F (dfg, dfe)*^p^*^-Value^	337.7 (2, 82.3)***	S: 14.9 (2, 79.0)*** L: 41.1 (2, 77.6)***	-	-	-	S: 58.9 (2, 75.6)*** L: 36.6 (2, 72.8)***

^1^ deletion of about 36% of 1RS arm (proximal part).

**Table 5:** Numbers of the G_1_, S, and G_2_ phase nuclei with complete separation, partial association and complete association of introgressed chromosomes or chromosome arms in various tissues of wheat-rye and wheat-barley introgression lines. Unless otherwise stated, the nuclei were in G_1_ phase and isolated from root tips.

Introgression	Complete Separation	Partial Association	Complete Association
1AS.1RL (embryo, G_1_)	19	1	2
1AS.1RL (leaf, G_1_)	16	4	1
1AS.1RL (root tip, G_1_)	22	3	
1AS.1RL (root tip, S)	18	3	
1AS.1RL (root tip, G_2_)	20	2	1
1RS.1BL	32	7	1
1RS.1DL	37	3	
2RS.2BL	32	8	
2BS.2RL	33	6	1
5RS.5BL	31	7	2
_del_1RS.1RL ^1^ (embryo, G_1_)	16	4	2
_del_1RS.1RL ^1^ (leaf, G_1_)	17		3
_del_1RS.1RL ^1^ (root tip, G_1_)	22	3	
_del_1RS.1RL ^1^ (root tip, S)	21	1	1
_del_1RS.1RL ^1^ (root tip, G_2_)	15	6	1
1RS._del_1RL ^2^	17	3	1
1R(1A) (G_1_)	15	5	2
1R(1A) (S)	15	4	1
1R(1A) (G_2_)	18	2	2
t1RS (G_1_)	20	1	1
t1RS (S)	20	1	
t1RS (G_2_)	19	2	2
3B	20		
5B	18	2	
3H	14	3	3
3HL	18	4	2
3HS	18	2	2
6H	17	2	1
6HL	14	4	2
6HS	19	1	

^1^ deletion of proximal about 36% of 1RS arm; ^2^ deletion of proximal about 30% of 1RL arm.

## Supplementary Materials

Supplementary materials can be found at https://www.mdpi.com/1422-0067/20/17/4143/s1. Video S1. Nucleus of hexaploid triticale with fluorescently labelled rye and wheat chromatin. Total genomic DNA of rye was labelled with FITC using Nick translation (green color), total genomic DNA of wheat was labelled with TRITC (yellow color), and centromeres of both wheat and rye chromosomes were visualized using oligonucleotide probe (magenta color). Nuclear DNA was counterstained with DAPI (blue color). Wheat and rye chromatin was visualized in Imaris 9.2. using ‘Surfaces’ function.

## Abbreviations

3D-FISH	Three-dimensional fluorescent in situ hybridization
CN	Centre of the nucleus
CT	Chromosome territory
GISH	Genomic in situ hybridization
MA	Arm mid-point
NOR	Nucleolar organizing region

## References

[1] Cremer T., Cremer C. (2001). Chromosome territories, nuclear architecture and gene regulation in mammalian cells. Nat. Rev. Genet..

[2] Fritz A.J., Barutcu A.R., Martin-Buley L., van Wijnen A.J., Zaidi S.K., Imbalzano A.N., Lian J.B., Stein J.L., Stein G.S. (2016). Chromosomes at work: Organization of chromosome territories in the interphase nucleus. J. Cell. Biochem..

[3] Sun F.L., Cuaycong M.H., Craig C.A., Wallrath L.L., Locke J., Eigin S.C.R. (2000). The fourth chromosome of Drosophila melanogaster: Interspersed euchromatic and heterochromatic domains. Proc. Natl. Acad. Sci. USA.

[4] Kozubek S., Lukasova E., Jirsova P., Koutna I., Kozubek M., Ganova A., Bartova E., Falk M., Pasekova R. (2002). 3D Structure of the human genome: order in randomness. Chromosoma.

[5] Mayer R., Brero A., von Hase J., Schroeder T., Cremer T., Dietzel S. (2005). Common themes and cell type specific variations of higher order chromatin arrangements in the mouse. Bmc Cell Biol..

[6] Gorkin D.U., Leung D., Ren B. (2014). The 3D genome in transcriptional regulation and pluripotency. Cell Stem Cell.

[7] Croft J.A., Bridger J.M., Boyle S., Perry P., Teague P., Bickmore W.A. (1999). Differences in the localization and morphology of chromosomes in the human nucleus. J. Cell Biol..

[8] Cremer M., von Hase J., Volm T., Brero A., Kreth G., Walter J., Fischer C., Solovei I., Cremer C., Cremer T. (2001). Non-random radial higher-order chromatin arrangements in nuclei of diploid human cells. Chromosome Res..

[9] Tanabe H., Muller S., Neusser M., von Hase J., Calcagno E., Cremer M., Solovei I., Cremer C., Cremer T. (2002). Evolutionary conservation of chromosome territory arrangements in cell nuclei from higher primates. Proc. Natl. Acad. Sci. USA.

[10] Habermann F.A., Cremer M., Walter J., Kreth G., von Hase J., Bauer K., Wienberg J., Cremer C., Cremer T., Solovei I. (2001). Arrangements of macro- and microchromosomes in chicken cells. Chromosome Res..

[11] Manvelyan M., Hunstig F., Bhatt S., Mrasek K., Pellestor F., Weise A., Simonyan I., Aroutiounian R., Liehr T. (2008). Chromosome distribution in human sperm—A 3D multicolor banding-study. Mol. Cytogenet..

[12] Manvelyan M., Hunstig F., Mrasek K., Bhatt S., Pellestor F., Weise A., Liehr T. (2008). Position of chromosomes 18, 19, 21 and 22 in 3D-preserved interphase nuclei of human and gorilla and white hand gibbon. Mol. Cytogenet..

[13] Rabl C. (1885). Über Zellteilung. Morph. Jahrb..

[14] Dong F.G., Jiang J.M. (1998). Non-Rabl patterns of centromere and telomere distribution in the interphase nuclei of plant cells. Chromosome Res..

[15] Schubert I., Shaw P. (2011). Organization and dynamics of plant interphase chromosomes. Trends Plant Sci..

[16] Pecinka A., Schubert V., Meister A., Kreth G., Klatte M., Lysak M.A., Fuchs J., Schubert I. (2004). Chromosome territory arrangement and homologous pairing in nuclei of Arabidopsis thaliana are predominantly random except for NOR-bearing chromosomes. Chromosoma.

[17] Fransz P., de Jong J.H., Lysak M., Castiglione M.R., Schubert I. (2002). Interphase chromosomes in Arabidopsis are organized as well defined chromocenters from which euchromatin loops emanate. Proc. Natl. Acad. Sci. USA.

[18] Schubert V., Rudnik R., Schubert I. (2014). Chromatin associations in arabidopsis interphase nuclei. Front. Genet..

[19] Berr A., Schubert I. (2007). Interphase chromosome arrangement in Arabidopsis thaliana is similar in differentiated and meristematic tissues and shows a transient mirror symmetry after nuclear division. Genetics.

[20] Dekker J., Rippe K., Dekker M., Kleckner N. (2002). Capturing chromosome conformation. Science.

[21] Lieberman-Aiden E., van Berkum N.L., Williams L., Imakaev M., Ragoczy T., Telling A., Amit I., Lajoie B.R., Sabo P.J., Dorschner M.O. (2009). Comprehensive mapping of long-range interactions reveals folding principles of the human genome. Science.

[22] Dixon J.R., Selvaraj S., Yue F., Kim A., Li Y., Shen Y., Hu M., Liu J.S., Ren B. (2012). Topological domains in mammalian genomes identified by analysis of chromatin interactions. Nature.

[23] Abney J.R., Cutler B., Fillbach M.L., Axelrod D., Scalettar B.A. (1997). Chromatin dynamics in interphase nuclei and its implications for nuclear structure. J. Cell Biol..

[24] Dong P., Tu X., Li H., Zhang J., Grierson D., Li P., Zhong S. (2019). Tissue-specific Hi-C analyses of rice, foxtail millet and maize suggest non-canonical function of plant chromatin domains. J. Intagr. Plant Biol..

[25] Doyle J.J., Flagel L.E., Paterson A.H., Rapp R.A., Soltis D.E., Soltis P.S., Wendel J.F. (2008). Evolutionary genetics of genome merger and doubling in plants. Annu. Rev. Genet..

[26] Schardin M., Cremer T., Hager H.D., Lang M. (1985). Specific staining of human-chromosomes in chinese-hamster *x* man hybrid cell-lines demonstrates interphase chromosome territories. Hum. Genet..

[27] Sengupta K., Camps J., Mathews P., Barenboim-Stapleton L., Nguyen Q.T., Difilippantonio M.J., Ried T. (2008). Position of human chromosomes is conserved in mouse nuclei indicating a species-independent mechanism for maintaining genome organization. Chromosoma.

[28] Heslopharrison J.S., Leitch A.R., Schwarzacher T., Anamthawatjonsson K. (1990). Detection and characterization of 1b/1r translocations in hexaploid wheat. Heredity.

[29] Kopecky D., Allen D.C., Duchoslav M., Dolezel J., Lukaszewski A.J. (2007). Condensation of rye chromatin in somatic interphase nuclei of Ph1 and ph1b wheat. Cytogenet. Genome Res..

[30] Schlegel R., Melz G., Nestrowicz R. (1987). A Universal reference karyotype in rye, *Secale cereale* L.. Theor. Appl. Genet..

[31] Naranjo T. (2018). Variable patterning of chromatin remodeling, telomere positioning, synapsis, and chiasma formation of individual rye chromosomes in meiosis of wheat-rye additions. Front. Plant Sci..

[32] Gill B.S., Friebe B., Endo T.R. (1991). Standard karyotype and nomenclature system for description of chromosome bands and structural-aberrations in wheat (*Triticum aestivum*). Genome.

[33] Paux E., Sourdille P., Salse J., Saintenac C., Choulet F., Leroy P., Korol A., Michalak M., Kianian S., Spielmeyer W. (2008). A physical map of the 1-gigabase bread wheat chromosome 3B. Science.

[34] Salina E.A., Nesterov M.A., Frenkel Z., Kiseleva A.A., Timonova E.M., Magni F., Vrana J., Safar J., Simkova H., Dolezel J. (2018). Features of the organization of bread wheat chromosome 5BS based on physical mapping. Bmc Genom..

[35] Mascher M., Gundlach H., Himmelbach A., Beier S., Twardziok S.O., Wicker T., Radchuk V., Dockter C., Hedley P.E., Russell J. (2017). A chromosome conformation capture ordered sequence of the barley genome. Nature.

[36] Dolezel J., Greilhuber J., Lucretti S., Meister A., Lysak M.A., Nardi L., Obermayer R. (1998). Plant genome size estimation by flow cytometry: Inter-laboratory comparison. Ann. Bot..

[37] Sequeira-Mendes J., Gutierrez C. (2016). Genome architecture: from linear organisation of chromatin to the 3D assembly in the nucleus. Chromosoma.

[38] Abranches R., Beven A.F., Aragon-Alcaide L., Shaw P.J. (1998). Transcription sites are not correlated with chromosome territories in wheat nuclei. J. Cell Biol..

[39] Mikhailova E.I., Sosnikhina S.P., Kirillova G.A., Tikholiz O.A., Smirnov V.G., Jones R.N., Jenkins G. (2001). Nuclear dispositions of subtelomeric and pericentromeric chromosomal domains during meiosis in asynaptic mutants of rye (Secale cereale L.). J. Cell Biol..

[40] Corredor E., Diez M., Shepherd K., Naranjo T. (2005). The positioning of rye homologous chromosomes added to wheat through the cell cycle in somatic cells untreated and treated with colchicine. Cytogenet. Genome Res..

[41] Idziak D., Robaszkiewicz E., Hasterok R. (2015). Spatial distribution of centromeres and telomeres at interphase varies among brachypodium species. J. Exp. Bot..

[42] Parada L.A., Misteli T. (2002). Chromosome positioning in the interphase, nucleus. Trends Cell Biol..

[43] Schwarzacher T., Leitch A.R., Bennett M.D., Heslop-Harrison J.S. (1989). In situ localization of parental genomes in a wide hybrid. Ann. Bot..

[44] Schubert I., Shi F., Fuchs J., Endo T.R. (1998). An efficient screening for terminal deletions and translocations of barley chromosomes added to common wheat. Plant J..

[45] Robaszkiewicz E., Idziak-Helmcke D., Tkacz M.A., Chrominski K., Hasterok R. (2016). The arrangement of *Brachypodium distachyon* chromosomes in interphase nuclei. J. Exp. Bot..

[46] Fung J.C., Marshall W.F., Dernburg A., Agard D.A., Sedat J.W. (1998). Homologous chromosome pairing in *Drosophila melanogaster* proceeds through multiple independent initiations. J. Cell Biol..

[47] Baroux C., Pecinka A., Fuchs J., Kreth G., Schubert I., Grossniklaus U. (2017). Non-random chromosome arrangement in triploid endosperm nuclei. Chromosoma.

[48] Hiraoka Y., Dernburg A.F., Parmelee S.J., Rykowski M.C., Agard D.A., Sedat J.W. (1993). The onset of homologous chromosome-pairing during *Drosophila melanogaster* embryogenesis. J. Cell Biol..

[49] Bornfleth H., Edelmann P., Zink D., Cremer T., Cremer C. (1999). Quantitative motion analysis of subchromosomal foci in living cells using four-dimensional microscopy. Biophys. J..

[50] Lucas J.N., Cervantes E. (2002). Significant large-scale chromosome territory movement occurs as a result of mitosis, but not during interphase. Int. J. Rad. Biol..

[51] Lam E., Kato N., Watanabe K. (2004). Visualizing chromosome structure/organization. Annu. Rev. Plant Biol..

[52] Kato N., Lam E. (2003). Chromatin of endoreduplicated pavement cells has greater range of movement than that of diploid guard cells in *Arabidopsis thaliana*. J. Cell Sci..

[53] Gartenberg M.R., Neumann F.R., Laroche T., Blaszczyk M., Gasser S.M. (2004). Sir-mediated repression can occur independently of chromosomal and subnuclear contexts. Cell.

[54] Tiang C.L., He Y., Pawlowski W.P. (2012). Chromosome organization and dynamics during Interphase, mitosis, and meiosis in plants. Plant Physiol..

[55] Soutoglou E., Misteli T. (2007). Mobility and immobility of chromatin in transcription and genome stability. Curr. Opin. Genet. Dev..

[56] Jasencakova Z., Meister A., Schubert I. (2001). Chromatin organization and its relation to replication and histone acetylation during the cell cycle in barley. Chromosoma.

[57] Bolzer A., Kreth G., Solovei I., Koehler D., Saracoglu K., Fauth C., Muller S., Eils R., Cremer C., Speicher M.R. (2005). Three-dimensional maps of all chromosomes in human male fibroblast nuclei and prometaphase rosettes. PLoS Biol..

[58] Bauer E., Schmutzer T., Barilar I., Mascher M., Gundlach H., Martis M.M., Twardziok S.O., Hackauf B., Gordillo A., Wilde P. (2017). Towards a whole-genome sequence for rye (*Secale cereale* L.). Plant J..

[59] Schwarzacher-Robinson T., Finch R.A., Smith J.B., Bennett M.D. (1987). Genotypic control of centromere positions of parental genomes in *Hordeum* x *Secale* hybrid metaphases. J. Cell Sci..

[60] Leitch A.R., Schwarzacher T., Mosgoller W., Bennett M.D., Heslop-Harrison J.S. (1991). Parental genomes are separated throughout the cell cycle in a plant hybrid. Chromosoma.

[61] Lukaszewski A.J. (2010). Behavior of Centromeres in Univalents and Centric Misdivision in Wheat. Cytogenet. Genome Res..

[62] Pernickova K., Kolackova V., Lukaszewski A.J., Fan C.L., Vrana J., Duchoslav M., Jenkins G., Phillips D., Samajova O., Sedlarova M. (2019). Instability of alien chromosome introgressions in wheat associated with improper positioning in the nucleus. Int. J. Mol. Sci..

[63] Ito H., Nasuda S., Endo T.R. (2004). A direct repeat sequence associated with the centromeric retrotransposons in wheat. Genome.

